# GenAI literacy and GenAI-assisted self-regulated learning behaviors: the roles of learning agency and challenge emotions

**DOI:** 10.3389/fpsyg.2026.1902668

**Published:** 2026-09-22

**Authors:** Juan Yang, Xiage Liu, Wei Zhao, Tingzhao Wang

**Affiliations:** 1 Faculty of Education, Shaanxi Normal University, Xi'an, China; 2 College of Special Education, Leshan Normal University, Leshan, China; 3 School of Teacher Education, Xianyang Polytechnic Institute, Xianyang, China

**Keywords:** challenge emotions, GenAI literacy, GenAI-assisted self-regulated learning behaviors, learning agency, special education undergraduates

## Abstract

The integration of generative artificial intelligence (GenAI) into education raises important questions about how learners' GenAI-related competencies are associated with their self-regulatory behaviors in GenAI-supported learning. Drawing on a serial mediation framework, this study examined the roles of learning agency and challenge emotions in the association between GenAI literacy and GenAI-assisted self-regulated learning behaviors among special education undergraduates. A cross-sectional survey was conducted with 434 special education undergraduates in China. Participants completed measures of GenAI literacy, learning agency, challenge emotions, and GenAI-assisted self-regulated learning behaviors. The results showed that GenAI literacy was positively associated with GenAI-assisted self-regulated learning behaviors. Significant indirect effects were observed through learning agency and challenge emotions separately, as well as through their hypothesized serial ordering. Overall, these findings suggest that both agentic and affective processes are relevant to the association between GenAI literacy and GenAI-assisted self-regulated learning behaviors and may also be interconnected.

## 1 Introduction

The rapid integration of generative artificial intelligence (GenAI) into education has transformed how learners access information, receive feedback, generate ideas, and engage with learning tasks (Kasneci et al., 2023; Li et al., 2025). Although GenAI can provide immediate information and cognitive support, learners remain responsible for directing and managing their own learning processes when using these tools. Self-regulated learning (SRL) is broadly understood as an active, goal-directed process through which learners regulate their cognition, motivation, affect, and behavior in relation to learning goals and contextual demands (Pintrich, 2004; Zimmerman, 2000). Contemporary reviews similarly emphasize that SRL encompasses cognitive, metacognitive, motivational, emotional, and behavioral aspects of learning (Panadero, 2017). Within this broader regulatory framework, learners may use a range of strategies to manage their attention, emotions, and actions in pursuing learning goals. These regulatory behaviors remain important when GenAI is incorporated into learning, as learners must continue to manage their own learning while interacting with AI-generated support. The present study refers to the regulatory behaviors examined in this context as GenAI-assisted self-regulated learning behaviors (SRLB).

GenAI is increasingly being explored in special education to support personalized instruction, adaptive interventions, intelligent assessment, and ongoing monitoring for students with disabilities (Hussein et al., 2025; Wei et al., 2025). As future special education professionals, special education undergraduates are expected to respond to diverse learner needs and complex instructional contexts (Harkins-Brown et al., 2025). As GenAI becomes increasingly embedded in these educational contexts, the ability to remain actively engaged and to regulate one's learning becomes increasingly important alongside the growing availability of AI-generated support. For special education undergraduates, examining SRLB in GenAI-supported environments can therefore provide insight into how they manage their learning while preparing for an increasingly AI-supported educational landscape.

Despite its potential to support learning, the growing use of GenAI has also raised concerns about how learners engage with these tools. Uncritical or excessive reliance on GenAI may encourage cognitive offloading and undermine learners' cognitive autonomy (Izquierdo-Condoy et al., 2025; Miranda et al., 2025). These concerns suggest that the educational value of GenAI may depend not simply on access to or frequency of use, but also on learners' capacity to use GenAI critically and appropriately and to regulate their own learning while doing so.

One factor that may be particularly relevant to such engagement is GenAI literacy. GenAI literacy can be conceptualized as a set of competencies for understanding, evaluating, and using generative AI effectively and responsibly (Annapureddy et al., 2025). Specifically, GenAI literacy encompasses knowledge of GenAI and its capabilities and limitations, the ability to evaluate AI-generated outputs, and skills for using GenAI appropriately across different contexts. Existing research on GenAI literacy has largely focused on its conceptualization, theoretical frameworks, assessment, and development (Sun and Zhou, 2025; Liu et al., 2025; Li et al., 2025). More recent studies have examined its associations with a range of learning-related outcomes, including academic achievement (Wang, 2026), GenAI-related learning behavior (Ma and Guo, 2026), problem-solving (Rodger et al., 2025), and innovative behavior (Weikang et al., 2025). However, less is known about how GenAI literacy is associated with learners' self-regulatory behaviors during GenAI-supported learning. Moreover, the potential roles of agentic and emotional factors in this association remain insufficiently understood.

To examine these potential roles, the present study draws on Social Cognitive Theory (Bandura, 2001) and Control–Value Theory of Achievement Emotions (Pekrun, 2006). Social Cognitive Theory provides a theoretical basis for considering learning agency, whereas Control–Value Theory provides a framework for understanding the role of emotional experiences in achievement-related activities. Together, these perspectives inform the hypothesized relationships among the study variables. Together, these perspectives provide a basis for examining how agentic and emotional experiences are associated with self-regulatory behavior during GenAI-supported learning. Building on this framework, the present study examines a hypothesized serial mediation model in which learning agency and challenge emotions are specified as sequential mediators in the association between GenAI literacy and GenAI-assisted self-regulated learning behaviors among special education undergraduates. By examining these hypothesized direct and indirect associations, the study seeks to extend understanding of how GenAI literacy is associated with self-regulatory behavior in GenAI-supported learning and may inform educational practices aimed at supporting more purposeful engagement with GenAI.

## 2 Literature review

### 2.1 GenAI literacy and GenAI-assisted self-regulated learning behaviors

GenAI literacy encompasses competencies for understanding the capabilities and limitations of GenAI, critically assessing AI-generated outputs, and using GenAI appropriately across different contexts (Annapureddy et al., 2025). These competencies may help learners make informed decisions about when and how to incorporate GenAI support into their learning while maintaining active regulation of their own learning. GenAI literacy may therefore be positively associated with GenAI-assisted self-regulated learning behaviors.

Recent studies have examined the relationship between AI literacy and self-regulated learning in different educational contexts. Using data from 11,020 Chinese K−12 students, Wang et al. (2026) found systematic differences in self-regulated learning across AI literacy profiles, with students in more advanced AI literacy profiles reporting stronger goal setting, goal pursuit, performance monitoring, and overall self-regulated learning. In a GenAI-supported learning context, Wang and Zhang (2026) found that the awareness and evaluation dimensions of GenAI literacy significantly predicted GenAI-supported self-regulated learning among Chinese university foreign language learners, although the associations varied across literacy dimensions. Similarly, Bećirović et al. (2026) found that AI technical understanding was positively associated with self-regulated learning among university students. These findings indicate that competencies related to understanding and evaluating AI are associated with learners' self-regulation in AI-supported learning. Learners with greater GenAI literacy may therefore be more likely to engage in self-regulatory behaviors when incorporating GenAI into their learning. Accordingly, we propose the following hypothesis:

*H1. GenAI literacy is positively associated with GenAI-assisted self-regulated learning behaviors among special education undergraduates*.

### 2.2 The mediating role of learning agency

In GenAI-supported learning, learning agency refers to learners' capacity to make choices and take actions in directing their learning, together with the abilities and mental processes involved in these activities (Xia et al., 2025a). GenAI literacy may be relevant to the exercise of learning agency because competencies for understanding, evaluating, and appropriately using GenAI can help learners make more informed choices about their engagement with AI during learning. Lim and Teh (2026) found that AI literacy was positively associated with student learning agency in AI-enhanced online learning. Related evidence also indicates that students' patterns of GenAI use are associated with their learning agency (Xia et al., 2025a).

The exercise of learning agency, in turn, may be relevant to how learners regulate their actions in pursuit of learning goals. From the perspective of Social Cognitive Theory, Bandura (2001) characterized human agency in terms of intentionality, forethought, self-reactiveness, and self-reflectiveness. Through these agentic processes, individuals intentionally pursue anticipated goals, regulate their actions, and reflect on their own functioning. Educational research has also highlighted the close relationship between learner agency and self-regulation. Mairitsch et al. (2023) described learner agency and self-regulation as interconnected, noting that learners who feel agentic in their learning can exercise self-regulatory strategies and behaviors. A recent scoping review similarly reported that learners who perceive themselves as agents are more likely to use self-regulatory strategies effectively (Gupta et al., 2024). In GenAI-supported learning, stronger learning agency may therefore be associated with greater engagement in self-regulatory behaviors while learners draw on GenAI support. Taken together, these relationships suggest that learning agency may mediate the association between GenAI literacy and GenAI-assisted self-regulated learning behaviors. Accordingly, we propose the following hypothesis:

*H2. Learning agency mediates the association between GenAI literacy and GenAI-assisted self-regulated learning behaviors*.

### 2.3 The mediating role of challenge emotions

Challenge emotions arise when individuals perceive an event as an opportunity and have some perceived control over its consequences (Beaudry and Pinsonneault, 2010; Yin et al., 2024). They include affective experiences such as excitement, playfulness, arousal, and flow. This appraisal-based view is consistent with Control–Value Theory, which emphasizes the role of learners' appraisals in shaping emotional experiences during learning activities and outcomes (Pekrun, 2006). In GenAI-supported learning, GenAI literacy may also be associated with learners' emotional experiences during their engagement with GenAI. Recent research has begun to examine this relationship. For example, Xie et al. (2026) found that some dimensions of AI literacy were positively associated with enjoyment during AI-mediated teaching. Greater GenAI literacy may therefore be associated with more positive challenge emotions during GenAI-supported learning.

Challenge emotions, in turn, may be relevant to how learners regulate their learning. Research on emotions in learning suggests that positive emotional experiences are associated with learners' use of self-regulatory learning strategies. Prior research has shown that enjoyment was positively associated with elaboration and metacognition among undergraduates in an online learning environment (Artino and Jones, 2012). A critical review and meta-analysis of 23 empirical studies similarly identified systematic associations between academic emotions and self-regulated learning strategies (Zheng et al., 2023). In GenAI-supported learning, challenge emotions may therefore be associated with more active use of self-regulatory strategies while learners engage with GenAI. Taken together, these relationships suggest that challenge emotions may mediate the association between GenAI literacy and GenAI-assisted self-regulated learning behaviors. Accordingly, we propose the following hypothesis:

*H3. Challenge emotions mediate the association between GenAI literacy and GenAI-assisted self-regulated learning behaviors*.

### 2.4 The serial mediating role of learning agency and challenge emotions

Learning agency reflects learners' capacity to intentionally regulate and direct their learning processes toward desired goals (Vaughn, 2018; Huo, 2024). From the perspective of Social Cognitive Theory, human agency is characterized by intentionality, forethought, self-reactiveness, and self-reflectiveness (Bandura, 2001). Such agentic involvement may also be relevant to how learners appraise the learning situations they encounter. According to the Control–Value Theory of Achievement Emotions, learners' appraisals of learning activities and outcomes play a central role in shaping their emotional experiences (Pekrun, 2006). Consistent with this proposed relationship, Chen et al. (2025) found that learner agency and emotions were dynamically interconnected, with stronger agency being associated with more positive emotional experiences. In GenAI-supported learning, stronger learning agency may therefore be associated with greater challenge emotions during learners' engagement with GenAI. Building on the relationships proposed above, we propose the following hypothesis:

*H4. Learning agency and challenge emotions serially mediate the association between GenAI literacy and GenAI-assisted self-regulated learning behaviors*.

Taken together, the proposed relationships form a serial mediation model in which learning agency and challenge emotions are specified as sequential mediators in the association between GenAI literacy and GenAI-assisted self-regulated learning behaviors (Figure 1).

**Figure 1:**
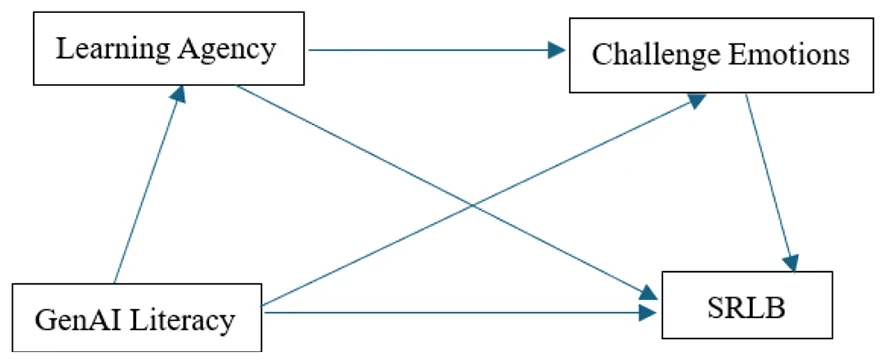
Hypothesized model. SRLB, GenAI-assisted self-regulated learning behaviors.

## 3 Methods

### 3.1 Participants and procedure

This study employed a convenience sampling method and conducted a cross-sectional online survey among special education undergraduates from universities in Shaanxi, Sichuan, Yunnan, and Hainan provinces in China. Data were collected between May and June 2025. These universities were selected because they offered special education programs and were accessible through the research team's existing academic collaborations. Eligibility criteria required participants to be full-time undergraduate students majoring in special education who had prior experience using GenAI tools.

Before the formal survey, participants were informed about the purpose of the study and assured that their participation was voluntary and that all responses would remain anonymous and confidential. Only those who provided informed consent proceeded to the survey. The questionnaire was distributed online through Wenjuanxing and shared via WeChat. Participants completed the questionnaire independently and were encouraged to respond based on their actual learning experiences. A total of 503 questionnaires were initially collected. To ensure data quality, responses with obvious response patterns, excessive missing values, or unusually short completion times were excluded. After screening, 434 valid responses were retained for analysis, representing 86.3% of the questionnaires initially collected. The final sample consisted of 396 female students (91.2%) and 38 male students (8.8%). Although female participants substantially outnumbered male participants, this distribution is consistent with that reported for special education undergraduates in China in previous studies (Li, 2018; Liu, 2021). Regarding academic year, 77 participants (17.7%) were first-year students, 79 (18.2%) were second-year students, 261 (60.1%) were third-year students, and 17 (3.9%) were fourth-year students.

The survey included measures of GenAI literacy, learning agency, challenge emotions, and GenAI-assisted self-regulated learning behaviors, together with demographic information. The original instruments used to measure learning agency, challenge emotions, and GenAI-assisted self-regulated learning behaviors were developed in English and were translated into Chinese and back-translated by bilingual researchers. Minor revisions were made following discussions among the research team to improve clarity and ensure conceptual equivalence across language versions.

### 3.2 Measurement instruments

#### 3.2.1 Generative artificial intelligence literacy scale

Based on the characteristics of generative artificial intelligence and expert consultation, items from the College Students' Artificial Intelligence Literacy Scale developed by Zhou et al. (2024a) were adapted to develop a Generative Artificial Intelligence Literacy Scale tailored for special education undergraduates. The final scale consists of 25 items across four dimensions: GenAI knowledge, skills, attitudes and values, and ethics. All items were rated on a 7-point Likert scale, with higher scores indicating higher levels of GenAI literacy. In the present study, Cronbach's α was 0.885.

#### 3.2.2 Learning agency

Students' learning agency in GenAI-supported contexts was measured using the Student Learning Agency Scale in Generative Artificial Intelligence-Supported Contexts developed Xia et al. (2025b). The scale consists of 34 items across 10 factors, which are organized into three broader dimensions: key ability, active action, and essential mental characteristics. Responses were rated on a 5-point Likert scale, with higher scores indicating stronger learning agency in GenAI-supported contexts. In the present study, Cronbach's α was 0.942.

#### 3.2.3 Challenge emotions

Challenge emotions were measured using four items adapted from Yin et al. (2024) to assess emotional experiences during GenAI use. The items assessed playfulness, flow, excitement, and arousal. Participants rated each item on a 5-point Likert scale, with higher scores indicating higher levels of challenge emotions. In the present study, Cronbach's α was 0.865.

#### 3.2.4 GenAI-assisted self-regulated learning behaviors

GenAI-assisted self-regulated learning behaviors (SRLB) were assessed using 11 items adapted by Zhou et al. (2024b) from Rosenbaum (1980). Self-Control Schedule for GenAI context. In the present study, these items were conceptualized as reflecting learners' enactment of self-regulatory strategies when using GenAI to support their learning. Participants rated each item on a 7-point Likert scale, with higher scores indicating greater engagement in GenAI-assisted self-regulated learning behaviors. In the present study, Cronbach's α was 0.911.

### 3.3 Data analysis

Data were analyzed using R. Descriptive statistics, internal consistency reliability, and bivariate correlations were examined. Potential common method variance was assessed using an item-level common latent method factor model, in which a common method factor was specified in addition to the substantive factors. Harman's single-factor test was retained as a supplementary diagnostic. The distinctiveness of the four factor constructs was evaluated using confirmatory factor analysis (CFA). In the hypothesized four-factor measurement model, GenAI literacy was represented by its four subscale scores, learning agency by its ten subscale scores, challenge emotions by its four items, and GenAI-assisted self-regulated learning behaviors by its 11 items. The measurement model was estimated using robust maximum likelihood (MLR) and compared with alternative three-factor models in which theoretically adjacent constructs were combined. Discriminant validity was further assessed using the heterotrait–monotrait ratio (HTMT).

Gender and year of study were included as covariates in the mediation analyses. Multicollinearity was assessed using variance inflation factors (VIFs) and tolerance values, and heteroskedasticity was examined using the Breusch–Pagan test. When evidence of heteroskedasticity was detected, HC3 robust standard errors were additionally examined as a sensitivity analysis. The hypothesized serial mediation model was estimated using the lavaan package with 5,000 bootstrap resamples. Indirect effects were evaluated using 95% bootstrap confidence intervals, with intervals excluding zero indicating statistical significance.

## 4 Results

### 4.1 Common method bias and regression diagnostics

Given that the data were obtained via student self-reports, the potential for common method bias was examined. Following Zhou and Long (2004), Harman's single-factor test was first conducted. The unrotated factor solution yielded 14 factors with eigenvalues >1, with the first factor accounting for 25.747% of the total variance, below the commonly used 40% threshold. To provide a more stringent assessment of potential common method variance, a common latent method factor (CLF) model was subsequently estimated. The CLF analysis indicated the presence of some method-related variance; however, the average variance attributable to the method factor across the 74 items was 4.86% (median = 3.38%). Importantly, the substantive factor loadings remained significant after inclusion of the method factor, and the key associations between GenAI literacy and learning agency and between learning agency and SRLB remained comparable in the models with and without the method factor. Taken together, these findings suggest that although some common method variance was present, it was unlikely to account for the key relationships among the study constructs.

In addition, diagnostic tests for multicollinearity and heteroscedasticity were conducted prior to the mediation analysis. Multicollinearity was assessed using variance inflation factor (VIF) and tolerance values. The VIF values ranged from 1.079 to 1.934, and the tolerance values ranged from 0.517 to 0.927, indicating that no problematic multicollinearity was detected among the predictors (Hair et al., 2010).

Homoscedasticity was assessed using the Breusch–Pagan test. No significant heteroscedasticity was detected for the learning agency, BP(5) = 5.77, *p* = 0.329, or challenge emotions equation, BP(6) = 5.41, *p* = 0.492, whereas heteroscedasticity was detected for the SRLB equation, BP(7) = 28.27, *p* < 0.001. A sensitivity analysis using HC3 robust standard errors yielded substantively unchanged results for the focal predictors of SRLB (GenAI literacy: *B* = 0.219, SEHC3 = 0.081, *p* = 0.007; learning agency: *B* = 0.653, SEHC3 = 0.108, *p* < 0.001; challenge emotions: *B* = 0.372, SEHC3 = 0.049, *p* < 0.001).

### 4.2 Descriptive statistics and correlation analysis

Table 1 presents the descriptive statistics, skewness and kurtosis values, and correlations among the study variables. The skewness values of the continuous variables ranged from −0.20 to 0.15, and the kurtosis values ranged from −0.19 to 0.84, indicating that all variables showed acceptable distributions. Gender was positively associated with year of study and learning agency but negatively associated with challenge emotions. GenAI literacy, learning agency, and challenge emotions were all positively associated with SRLB. Moreover, GenAI literacy was positively associated with both learning agency and challenge emotions, and learning agency was also positively associated with challenge emotions.

**Table 1:** Descriptive statistics and correlations among main variables (*n* = 434).

Variable	Skewness/Kurtosis	*M (SD)*	1	2	3	4	5	6
1.Gender	—	1.91 (0.28)	1					
2. Year of study	—	2.50 (0.83)	0.099^*^	1				
3. GenAI literacy	0.15; −0.07	5.33 (0.51)	0.003	−0.018	1			
4.Learning agency	−0.10;0.50	3.94 (0.40)	0.116^*^	0.019	0.671^***^	1		
5. Challenge emotions	−0.20;0.84	3.33 (0.66)	−0.105^*^	−0.070	0.282^***^	0.300^***^	1	
6. SRLB	0.09; −0.19	4.93 (0.75)	−0.058	−0.018	0.472^***^	0.535^***^	0.479^***^	1

SRLB, GenAI-assisted self-regulated learning behaviors. ^*^p < 0.05;^**^p < 0.01;^***^p < 0.001.

### 4.3 Measurement model and discriminant validity

The empirical distinctiveness of the four focal constructs was examined using a four-factor measurement model and a series of alternative models in which theoretically related constructs were combined. The four-factor measurement model yielded CFI = 0.852, TLI = 0.838, RMSEA = 0.077, and SRMR = 0.075. Although the incremental fit indices were below conventional benchmarks, the four-factor measurement model consistently provided a better representation of the data than the alternative merged models (Table 2).

**Table 2:** Comparison of the hypothesized four- factor model and alternative merged models.

Model	χ^2^	df	CFI	TLI	RMSEA	SRMR	Δχ^2^ (Δdf)	*p*
Four-factor model	1,067.417	371	0.852	0.838	0.077	0.075	—	—
GL + LA merged	1,108.374	374	0.844	0.830	0.079	0.077	38.01 (3)	< 0.001
LA + CE merged	1,605.039	374	0.736	0.714	0.102	0.103	284.23 (3)	< 0.001
CE + SRLB merged	1,469.194	374	0.765	0.745	0.096	0.088	205.18 (3)	< 0.001
GL + SRLB merged	1,317.338	374	0.799	0.782	0.089	0.094	215.28 (3)	< 0.001
LA + SRLB merged	1,697.434	374	0.716	0.692	0.106	0.099	297.31 (3)	< 0.001

GL, GenAI literacy; LA, learning agency; CE, challenge emotions; SRLB, GenAI-assisted self-regulated learning behaviors. χ^2^ values are scaled chi-square statistics. Δχ^2^ values are based on robust scaled chi-square difference tests comparing each merged model with the hypothesized four-factor model.

Specifically, combining GenAI literacy and learning agency significantly worsened model fit, Δχ^2^(3) = 38.01, *p* < 0.001. Similarly, models combining learning agency with challenge emotions, Δχ^2^(3) = 284.23, *p* < 0.001; challenge emotions with SRLB, Δχ^2^(3) = 205.18, *p* < 0.001; GenAI literacy with SRLB, Δχ^2^(3) = 215.28, *p* < 0.001; or learning agency with SRLB, Δχ^2^(3) = 297.31, *p* < 0.001, all fit significantly worse than the four-factor model.

Discriminant validity was further examined using the heterotrait–monotrait ratio (HTMT). The HTMT values were 0.767 for GenAI literacy–learning agency, 0.313 for GenAI literacy–challenge emotions, 0.516 for GenAI literacy–SRLB, 0.328 for learning agency–challenge emotions, 0.597 for learning agency–SRLB, and 0.538 for challenge emotions–SRLB. All values were below the conservative 0.85 threshold, providing additional evidence for the empirical distinctiveness of the four constructs.

Taken together, the competing-model comparisons and HTMT results supported the empirical distinguishability of the four focal constructs, although the absolute fit of the four-factor model was less than optimal.

### 4.4 Serial mediation analysis

The hypothesized serial mediation model was examined with GenAI literacy as the independent variable, learning agency and challenge emotions as sequential mediators, and GenAI-assisted self-regulated learning behaviors (SRLB) as the dependent variable. Gender and year of study were included as covariates. Indirect effects were evaluated using 5,000 bootstrap resamples. A summary of the hypothesis testing results is presented in Table 3, and the detailed regression results are presented in Tables 4, 5 presents the bootstrap indirect effects.

**Table 3:** Summary of hypothesis testing results.

Hypothesis	Hypothesis statement	Result
H1	GenAI literacy is positively associated with GenAI-assisted self-regulated learning behaviors among special education undergraduates.	Supported
H2	Learning agency mediates the association between GenAI literacy and GenAI-assisted self-regulated learning behaviors.	Supported
H3	Challenge emotions mediate the association between GenAI literacy and GenAI-assisted self-regulated learning behaviors.	Supported
H4	Learning agency and challenge emotions serially mediate the association between GenAI literacy and GenAI-assisted self-regulated learning behaviors.	Supported

**Table 4:** Regression analysis of variables in the model (*n* = 434).

Outcome	Predictor	*B*	*SE*	β	*p*
Learning agency	GenAI literacy	0.529	0.028	0.671	< 0.001
Challenge emotions	GenAI literacy	0.165	0.083	0.126	0.048
Challenge emotions	Learning agency	0.380	0.107	0.230	< 0.001
SRLB	GenAI literacy	0.219	0.079	0.148	0.006
SRLB	Learning agency	0.653	0.103	0.346	< 0.001
SRLB	Challenge emotions	0.372	0.047	0.326	< 0.001

Gender and year of study were included as covariates in all relevant equations but are omitted from the table for parsimony. SRLB, GenAI-assisted self-regulated learning behaviors.

**Table 5:** Mediating effect analysis of the serial mediating model.

Pathway	*B*	Boot SE	95% Boot CI	β
Total effect	0.701	0.067	[0.564, 0.830]	0.471
Direct effect	0.219	0.079	[0.062, 0.376]	0.148
Total indirect effect	0.481	0.064	[0.359, 0.607]	0.324
GenAI literacy → Learning agency → SRLB	0.345	0.058	[0.234, 0.461]	0.232
GenAI literacy → Challenge emotions → SRLB	0.061	0.032	[0.001, 0.126]	0.041
GenAI literacy → Learning agency → Challenge emotions → SRLB	0.075	0.023	[0.030, 0.122]	0.050

B, unstandardized effect; β, standardized effect; Boot SE, bootstrap standard error; Boot CI, bootstrap confidence interval. Bootstrap confidence intervals were based on 5,000 resamples. Indirect effects were considered statistically significant when the 95% bootstrap confidence interval did not include zero.

As shown in Table 4, GenAI literacy was significantly and positively associated with learning agency (*B* = 0.529, SE = 0.028, β = 0.671, *p* < 0.001). GenAI literacy also showed a small positive association with challenge emotions after accounting for learning agency and the covariates (*B* = 0.165, SE = 0.083, β = 0.126, *p* = 0.048), while learning agency was positively associated with challenge emotions (*B* = 0.380, SE = 0.107, β = 0.230, *p* < 0.001). GenAI literacy was positively associated with SRLB (*B* = 0.219, SE = 0.079, β = 0.148, *p* =0.006). Learning agency (*B* = 0.653, SE = 0.103, β = 0.346, *p* < 0.001) and challenge emotions (*B* = 0.372, SE = 0.047, β = 0.326, *p* < 0.001) were also positively associated with SRLB (see Figure 2).

**Figure 2:**
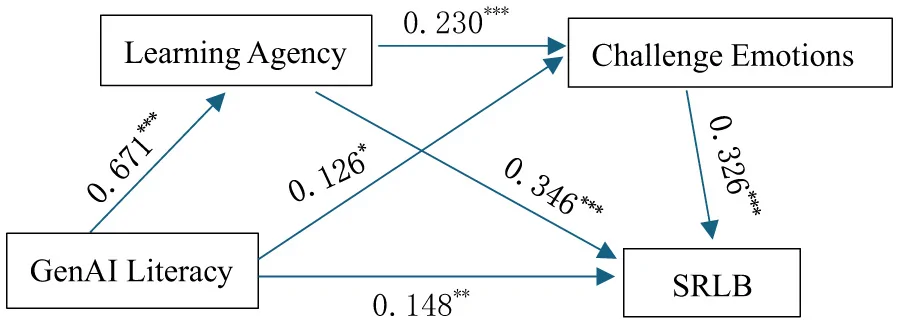
The serial mediation model. SRLB, GenAI-assisted self-regulated learning behaviors.

Bootstrap analyses with 5,000 resamples were conducted to evaluate the indirect effects. The indirect association of GenAI literacy with SRLB through learning agency was statistically significant (*B* = 0.345, SE = 0.058, 95% bootstrap CI [0.234, 0.461], β = 0.232). The indirect association through challenge emotions were also statistically significant, although relatively small in magnitude (*B* = 0.061, SE = 0.032, 95% bootstrap CI [0.001, 0.126], β = 0.041). In addition, the serial indirect association through learning agency and challenge emotions were statistically significant (*B* = 0.075, SE = 0.023, 95% bootstrap CI [0.030, 0.122], β = 0.050). The total indirect effect was significant (*B* = 0.481, SE = 0.064, 95% bootstrap CI [0.359, 0.607], β = 0.324).

The direct association between GenAI literacy and SRLB remained statistically significant after accounting for the mediators (*B* = 0.219, SE = 0.079, 95% CI [0.062, 0.376], β = 0.148), indicating partial mediation. The total effect of GenAI literacy on SRLB was *B* = 0.701 (SE = 0.067, 95% CI [0.564, 0.830], β = 0.471) (see Table 5).

## 5 Discussion

### 5.1 The association between genai literacy and genai-assisted self-regulated learning behaviors

The findings supported Hypothesis 1, showing that GenAI literacy was positively associated with GenAI-assisted self-regulated learning behaviors among special education undergraduates. This finding is consistent with recent evidence linking AI literacy to self-regulated learning (Pan and Zhang, 2026; Wang et al., 2026). The present findings extend this association to special education undergraduates and to the regulatory behaviors they report when using GenAI to support their learning.

GenAI can provide immediate and adaptive support, but its outputs also need to be evaluated in relation to learners' goals and the demands of a particular task. Learners with greater GenAI literacy may be better able to recognize what GenAI can and cannot do, judge the credibility and usefulness of its outputs, and determine how such outputs should be used in their learning. These competencies **may** provide a basis for decisions about when and how to draw on GenAI support, whether its responses need to be checked or revised, and how to adjust subsequent learning activities. This interpretation is consistent with Anders and Dux Speltz (2025), who linked GenAI literacies with processes of planning, iterative adjustment, monitoring, and evaluation during GenAI-supported learning. Viewed in this way, GenAI literacy may provide an important resource on which learners draw when regulating their learning with GenAI. This may help explain why students with higher GenAI literacy in the present study also reported greater engagement in GenAI-assisted self-regulated learning behaviors.

### 5.2 The mediating role of learning agency

The significant indirect effect through learning agency provides a more nuanced understanding of the association between GenAI literacy and GenAI-assisted self-regulated learning behaviors. Rather than suggesting that GenAI-related competencies are automatically translated into self-regulatory behaviors, the findings indicate that how actively learners exercise agency in directing their learning may be an important part of this association. This interpretation is consistent with the agentic perspective of Social Cognitive Theory, which emphasizes individuals' active and intentional involvement in directing their actions toward desired goals (Bandura, 2001). In the context of GenAI-supported learning, possessing the competencies to understand, evaluate, and use GenAI may therefore be insufficient by itself; learners' exercise of agency may also be relevant to how these resources are incorporated into their learning.

This role of learning agency may be particularly relevant in GenAI-supported learning because GenAI simultaneously provides learners with greater support and greater discretion over how that support is used. Learners can decide when to consult GenAI, whether to accept or revise its outputs, and how much responsibility to retain for their own learning. Such discretion places greater importance on learners' active involvement in directing their own learning, rather than on the availability of GenAI support alone. Previous research has similarly emphasized the close interplay between learner agency and self-regulation in learning contexts (Mairitsch et al., 2023). The present findings extend this perspective by suggesting that, in GenAI-supported learning, agency may help account for variation in how actively learners regulate their learning.

It is also noteworthy that gender was significantly associated with learning agency in the present model. Female students reported higher levels of learning agency than male students after controlling for GenAI literacy and year of study. This difference may be considered in light of previous research suggesting more positive professional orientations among female students, including stronger professional identity and more positive attitudes toward the teaching profession (Chen and Yang, 2018; Erdamar et al., 2016). These findings may provide some context for the higher levels of learning agency observed among female students in the present study, although the underlying factors were not directly examined. However, given the predominantly female composition of the present sample (91.2%), the observed gender effect should be interpreted cautiously. Future studies with more gender-balanced samples are needed to examine whether this pattern generalizes across gender groups.

### 5.3 The mediating role of challenge emotions

The significant indirect effect through challenge emotions provides further evidence of the role of affective experiences in the association between GenAI literacy and GenAI-assisted self-regulated learning behaviors. According to Control–Value Theory, achievement emotions are shaped by learners' appraisals of achievement activities and outcomes, particularly appraisals of control and value (Pekrun, 2006). In GenAI-supported learning, learners with greater GenAI literacy may be better able to understand the capabilities and limitations of GenAI, evaluate its outputs, and use it appropriately. These competencies may contribute to more positive appraisals and emotional experiences when learners engage with GenAI-supported learning activities. Emerging empirical evidence provides some support for a connection between AI literacy and affective experiences: Xie et al. (2025) found significant relationships between dimensions of AI literacy and AI-induced emotions, while Deshen and Aharony (2026) reported that students' AI literacy was positively associated with both challenge appraisal and positive emotions toward AI.

The positive association between challenge emotions and GenAI-assisted self-regulated learning behaviors is consistent with evidence that positive activating emotions can support adaptive learning processes. Positive activating emotions have been associated with motivation, cognitive engagement, and the use of adaptive learning strategies (Pekrun et al., 2002). More directly, Artino and Jones (2012) found that enjoyment positively predicted elaboration and metacognition among undergraduate students in an online learning environment, providing evidence that positive achievement emotions are associated with self-regulatory processes. These findings are consistent with the significant indirect effect observed in the present study, suggesting that challenge emotions may represent an affective component of the association between GenAI literacy and GenAI-assisted self-regulated learning behaviors. The present findings extend this literature to GenAI-supported learning, where higher levels of challenge emotions were associated with greater engagement in GenAI-assisted self-regulated learning behaviors.

It is also noteworthy that gender was significantly associated with challenge emotions. Female students reported lower levels of challenge emotions than male students after controlling for other variables. This finding may reflect gender-related differences in prior digital technology experiences and technology-related confidence when engaging with emerging technologies such as GenAI (Cai et al., 2017; Hargittai and Shafer, 2006; He et al., 2026). Although these factors were not directly examined in the present study, they may provide some context for the observed gender difference. This finding should nevertheless be interpreted cautiously given the gender imbalance in the present sample.

### 5.4 The serial mediating effects of learning agency and challenge emotions

Beyond their individual mediating roles, learning agency and challenge emotions also formed a significant serial indirect pathway between GenAI literacy and GenAI-assisted self-regulated learning behaviors. This finding suggests that the agentic and affective processes examined in the present study may be interconnected rather than operating independently. Learners with greater GenAI literacy may be better positioned to exercise agency in directing their engagement with GenAI, and this more active involvement in learning may be associated with more positive emotional experiences. Chen et al. (2025) similarly reported a dynamic interconnection between learner agency and emotions. This interconnection may be particularly relevant in GenAI-supported learning, where learners have considerable discretion over how they engage with and respond to GenAI support.

From the perspective of Social Cognitive Theory, learners exercise agency by actively and intentionally directing their actions and making choices in pursuit of desired goals (Bandura, 2001). Such agentic involvement may be associated with a stronger sense of being able to respond to and influence the course of one's learning. Control–Value Theory further helps explain how this sense of influence may be related to learners' emotions. According to the theory, learners' perceived control over achievement activities and outcomes is a central appraisal underlying their emotional experiences (Pekrun, 2006). Thus, the experience of being able to respond to and influence one's learning through agentic involvement may be associated with more positive emotional experiences.

Taken together, the findings suggest that both agentic and affective processes are relevant to the association between GenAI literacy and GenAI-assisted self-regulated learning behaviors and that these processes may also be interconnected. Social Cognitive Theory provides a useful perspective for understanding the agentic processes involved in this association, whereas Control–Value Theory helps explain the role of emotional experiences in GenAI-supported learning. These perspectives thus offer different but related ways of interpreting the agentic and affective processes observed in the present study.

## 6 Implications and limitations

The findings have implications for understanding learning with GenAI. Both learning agency and challenge emotions were involved in the indirect association between GenAI literacy and GenAI-assisted self-regulated learning behaviors, highlighting the relevance of agentic and affective processes to learners' self-regulation in GenAI-supported learning. Moreover, the significant serial indirect effect suggests that these processes may be interconnected, with learning agency associated with challenge emotions in GenAI-supported learning. These findings extend research on GenAI literacy by drawing attention to the agentic and affective processes involved in learners' self-regulation when using GenAI.

The findings also have practical implications for the preparation of special education undergraduates. Developing GenAI literacy should go beyond teaching students how to operate GenAI tools and should include opportunities to understand their capabilities and limitations, critically evaluate their outputs, and make informed decisions about their use in learning. The findings regarding learning agency also point to the value of providing students with opportunities to make choices about how GenAI is incorporated into their learning, rather than positioning them as passive recipients of AI-generated support. Similarly, the findings regarding challenge emotions suggest that students' emotional experiences deserve attention when they engage with GenAI. Educators may therefore consider learning activities and guidance that support both meaningful learner involvement and positive emotional experiences in GenAI-supported learning.

Several limitations should be acknowledged. First, the sample was limited to special education undergraduates from four provinces in China (Shaanxi, Sichuan, Yunnan, and Hainan), which may restrict the generalizability of the findings. The sample was also predominantly female (91.2%), and although gender was statistically controlled, this imbalance may have reduced the precision of the estimated gender effects. Future studies with more gender-balanced samples are needed to further examine gender-related differences and the generalizability of the findings. Second, the cross-sectional design limits causal and temporal interpretations of the observed relationships and indirect effects. In particular, the serial ordering of learning agency and challenge emotions was theoretically specified rather than established by the cross-sectional data, which cannot by itself determine the temporal or causal ordering of these processes (Rohrer et al., 2022). Longitudinal or experimental studies are needed to examine the directionality and temporal ordering of these relationships. Third, all focal constructs were assessed using self-report measures within the same survey. Although supplementary analyses did not indicate substantial common method variance, common method bias and some conceptual and measurement overlap among GenAI literacy, learning agency, and GenAI-assisted self-regulated learning behaviors cannot be completely excluded. Future research could incorporate behavioral or learning-process data to further differentiate these constructs. Participants' frequency of GenAI use was also not assessed and warrants consideration in future research.

## 7 Conclusions

This study examined the association between GenAI literacy and GenAI-assisted self-regulated learning behaviors among special education undergraduates, with particular attention to the roles of learning agency and challenge emotions. The findings showed that GenAI literacy was positively associated with GenAI-assisted self-regulated learning behaviors. Significant indirect effects were observed through learning agency and challenge emotions separately, as well as through their hypothesized serial ordering. Together, these findings suggest that both agentic and affective processes are relevant to the association between GenAI literacy and GenAI-assisted self-regulated learning behaviors and may also be interconnected. The study thus contributes to a more nuanced understanding of the factors associated with self-regulatory behaviors in GenAI-supported learning among special education undergraduates.

## Data Availability

The raw data supporting the conclusions of this article will be made available by the authors, without undue reservation.
